# IVF outcomes in cancer survivors: lower ovarian response and early embryo development but comparable success in frozen embryo transfer cycles — a retrospective cohort study

**DOI:** 10.3389/fendo.2026.1898170

**Published:** 2026-08-26

**Authors:** Xiaoqian Zhou, Zhijie Hu, Yijun Zhang, Qiuju Chen, Chengrui Ye, Yuqi Zhang, Yanping Kuang, Xuefeng Lu

**Affiliations:** Department of Assisted Reproduction, Shanghai Ninth People’s Hospital, Shanghai Jiao Tong University School of Medicine, Shanghai, China

**Keywords:** assisted reproductive technology, cancer, chemotherapy, frozen-embryo transfer, radiotherapy

## Abstract

**Objective:**

To evaluate whether women who have undergone cancer chemotherapy or radiotherapy achieve assisted reproductive technology (ART) outcomes comparable to those of non-cancer patients with equivalent ovarian reserve, and to explore the association between ART outcomes and the interval after chemotherapy or radiotherapy.

**Design:**

Retrospective cohort study.

**Patients:**

32 cancer survivors who had received chemotherapy or radiotherapy and 160 matched control women had similar ovarian functions without malignancy.

**Main outcomes:**

Number of oocytes retrieved, embryo development, cumulative pregnancy rate and cumulative live birth rate.

**Result:**

This study performed inverse probability of treatment weighting (IPTW) to minimize potential confounding factors. After IPTW adjustment, the baseline characteristics were well balanced between the survivor and control groups. In the weighted regression analysis, the survivors exhibited a lower number of MII oocytes (β=-3.06 95%CI [-5.568, -0.555], *P* = 0.02), and a reduced high-quality embryo rate (β=-0.25 95%CI [-0.375, -0.128], *P* < 0.001). However, in the FET cycle, there was no difference in cumulative pregnancy rate and cumulative live birth rate and abortion rate. Besides, there was no strong relationship between the interval after chemo or radio therapy and ART outcomes.

**Conclusion:**

This study demonstrated that when women with a history of chemotherapy or radiotherapy had similar ovarian function with women who had not experienced cancer still exhibited reduced number of MII oocyte and lower high-quality embryo rate and there was no difference in FET outcomes between survivor and control group. We also found no significant difference in different treat-to- IVF/ICSI interval in FET cycle outcomes.

## Introduction

According to the WHO, over 35 million new cancer cases are predicted by 2050 (1).With ongoing advancements in cancer therapies, an increasing number of young female patients are achieving long-term survival through different kinds of cancer therapy (2). However, commonly used treatments such as chemotherapy and radiotherapy are associated with varying degrees of gonadotoxicity, which may result in diminished ovarian reserve, reduced oocyte number, and impaired reproductive outcomes (3, 4). As survival rates improve, a key concern for women of reproductive years is how the disease and its treatment may impact future fertility (5). Importantly, fertility preservation has become an essential component of survivorship care. Recent studies have shown that female cancer patients consider fertility preservation an important determinant of quality of life after treatment and emphasize the need for timely fertility counseling. In addition, cancer patients demonstrate strong support for biomedical research aimed at improving long-term outcomes after cancer therapy (6, 7).

Survivors who have undergone chemotherapy or radiotherapy are purported to face an increased risk of infertility. Assisted reproductive technologies (ART), including *in vitro* fertilization (IVF) and intracytoplasmic sperm injection (ICSI), are therefore recommended for women with cancer who are unable to conceive naturally after cancer treatment (8).

Existing studies have indicated that women who have undergone chemotherapy or radiotherapy exhibit significantly reduced success rates in ART treatment (9–11). However, the underlying mechanisms responsible for this decline remain incompletely understood. One central question is whether the reduction in ART success is primarily attributable to the direct effects of cancer therapy on oocyte quality, or whether it is mainly a consequence of diminished ovarian reserve. Previous studies demonstrated cancer treatments including chemotherapy and abdominal-pelvic radiation can rapidly accelerate the decline of a woman’s primordial follicles, or ovarian reserve, and result in immediate or premature ovarian failure (12). The observed reduction in ART outcomes may primarily reflect diminished ovarian reserve, potentially obscuring the independent effects of chemotherapy and radiotherapy on oocyte competence and overall reproductive potential.

Nevertheless, a critical knowledge gap persists regarding whether the intrinsic quality of oocytes retrieved from cancer survivors is also compromised. At the same time, human studies investigating the impact of chemotherapy or radiotherapy on embryo quality are very limited. Only one study has reported a decline in embryo quality following chemotherapy, but the sample size of the survivor group was merely four (10). Experimental evidence from animal models suggests that chemotherapeutic agents, such as alkylating compounds, may induce DNA damage, mitochondrial dysfunction, and epigenetic alterations in surviving oocytes, potentially affecting subsequent embryo competence and developmental potential (13). Human data, however, remain limited and inconclusive, partly due to the confounding influence of reduced ovarian reserve, which makes it difficult to isolate the direct impact of treatment on oocyte competence from the quantitative loss of follicles.

Therefore, the present study was designed to investigate whether previous exposure to chemotherapy or radiotherapy affects ART outcomes in cancer survivors undergoing IVF/ICSI treatment. Specifically, we aimed to determine whether patients who have undergone chemotherapy or radiotherapy achieve ART outcomes comparable to those of non-cancer patients when their ovarian reserve is equivalent and whether ART outcomes are related to the interval after chemotherapy or radiotherapy. Clarifying potential associations between anticancer treatment and ART outcomes may provide data to support fertility counseling and guide the design of future larger prospective studies investigating fertility preservation strategies for cancer survivors.

## Materials and methods

### Patients

**Figure 1:**
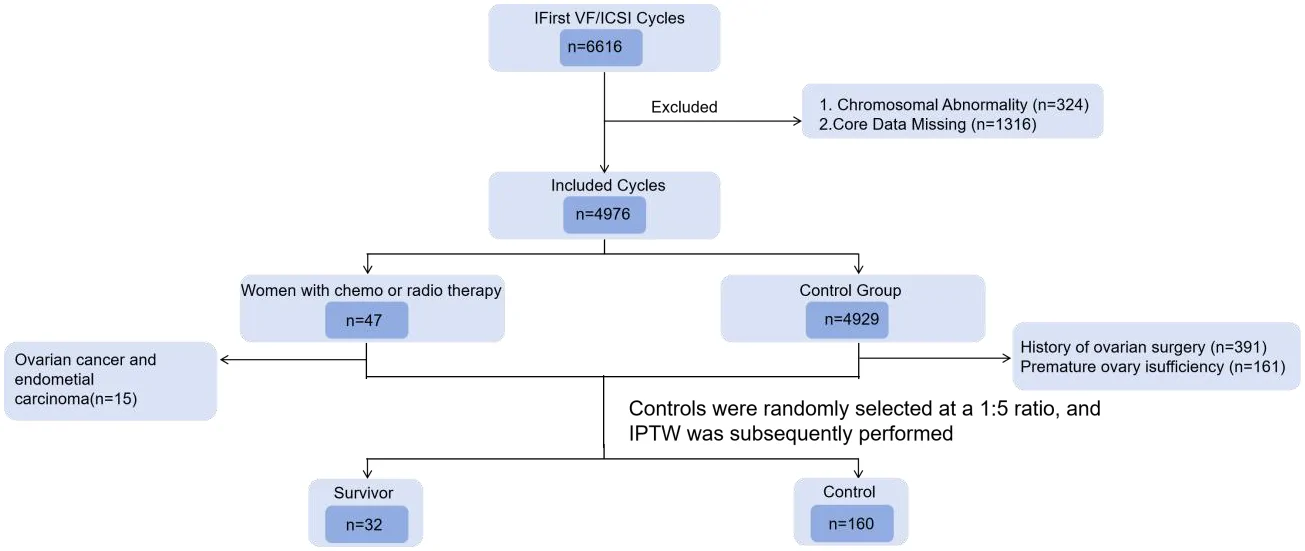
The flow chart of the study design and cohort selection.

This retrospective cohort study included women undergoing their first IVF/ICSI cycle at the Department of Assisted Reproduction of the Ninth People’s Hospital of Shanghai Jiao Tong University, between 2013 and 2024. A total of 6,616 IVF/ICSI cycles were initially identified. After excluding patients with chromosomal abnormalities or incomplete data, 4,976 cycles remained eligible for analysis (**Figure 1**).

The survivor group consisted of women with a history of malignancy who had previously received chemotherapy and/or radiotherapy and subsequently underwent IVF/ICSI, male patients with cancer or those who had received chemotherapy were not included. Clinical data, including cancer type, age at oncologic treatment, and treatment details, were extracted from medical records. To minimize confounding effects related to ovarian pathology, women with ovarian malignancy, prior ovarian surgery, or endometrial carcinoma were excluded. Women without malignancy and chemo/radio therapy undergoing IVF/ICSI at our hospital served as controls, with similar exclusion criteria applied, including prior ovarian surgery and premature ovarian insufficiency (POI).

Given the substantially larger number of controls, a random sampling strategy was applied to select control participants at a 1:5 ratio relative to the survivor group. A total of 160 control individuals were randomly selected and included in subsequent analyses. IPTW was then conducted based on the estimated propensity scores to further adjust for baseline differences between groups. Method is “cbps” and estimand is “ATE”. The propensity score model incorporated clinically relevant covariates, including age, BMI, FSH, E2, AFC, HMG dosage, HCG dosage, infertility duration, previous pregnancy history, and infertility etiology. After weighting, covariate balance between groups was assessed, and the weighted population was used for outcome comparisons, and the weighted population was used for outcome comparisons with further adjustment for relevant covariates.

Multivariate regression analysis was conducted to identify independent factors associated with high-quality embryo rate. Variables entered into the model included age, cancer therapy type, interval, body mass index (BMI), basal follicle stimulating hormone (FSH) level, basal estradiol(E_2_) level.

For FET cycles, IPTW was performed, with the following covariates included in the weighting model: age, BMI, infertile duration, number of previous pregnancies, basal FSH level, E2 level, endometrial thickness, number of embryos transferred, number of high-quality embryos transferred, and FET protocol.

### Ovarian stimulation protocol

The basal level of hormones was measured on the third day of menstruation. After 5 days, we monitored the follicles by transvaginal ultrasound. The serum luteinizing hormone (LH), FSH, E_2_ and progesterone (P) concentrations were measured on the same days as the ultrasound examinations. Thereafter, we monitored the follicles and sex hormone levels. When the diameter of two or more dominant follicles exceeded 18 mm, and the estradiol level was higher than 150 pg/ml per follicle, oocyte maturation was triggered by 0.1 mg triptorelin (Decapeptyl, Ferring Pharmaceuticals, Germany) and hCG (Lizhu Pharmaceutical Trading Co., China) (dual trigger). The oocytes were retrieved after 35–38 h.

### FET protocol

For mild stimulation FET cycles, letrozole 2.5 mg was administered for 3 days, when the follicle matured and endometrial thickness was ≥8 mm, hCG 5,000 IU was administered and cleavage embryo transfer was arranged 4–5 days later. The blastocyst transfer was arranged 2 days later.

For hormone replacement therapy, ethinyl estradiol 75 µg/day was orally administered from cycle day 3 onwards, when the endometrial lining was ≥8 mm thick, progesterone administration was started and cleavage embryos were transferred 3 days later.

### Outcomes

We compared IVF/ICSI outcomes including oocyte retrieval rate, number of mature oocytes, fertilization rate, and high-quality embryo rate as well as frozen embryo transfer (FET) outcomes such as cumulative pregnancy rate (CPR) and cumulative live birth rate (CLBR) and abortion rate. Additionally, we examined the relationship between interval and ART outcomes.

### Statistical analyses

Statistical analyses were performed using R software, version 4.2.2 (R Foundation for Statistical Computing, Vienna, Austria). Continuous variables were presented as mean ± standard Error of the Mean (SEM). Inverse probability of treatment weighting (IPTW) was applied to adjust for baseline differences between groups. Covariate balance after weighting was assessed using standardized mean differences (SMDs), with an SMD <0.1 considered indicative of adequate balance. Weighted regression models were subsequently performed to compare IVF outcomes between groups while accounting for the IPTW weights.

For cumulative pregnancy rate (CPR) and cumulative live birth rate (CLBR), IPTW-adjusted Kaplan-Meier analyses were performed, and differences between groups were evaluated using weighted log-rank tests. Weighted Cox proportional hazards models were additionally used to estimate 95% confidence intervals (95%CI).

### Ethics approval

The study protocol was approved by the institutional ethics committee of Shanghai Ninth People’s Hospital. All participants gave written informed consent after describing the research in detail.

## Results

### Patient characteristics

**Table 1** summarizes the baseline characteristics of the study population before and after IPTW. After matching, 32 women with a history of chemotherapy or radiotherapy for malignant tumors and 160 matched controls were included in the final analysis. Baseline characteristics were well balanced between the two groups, with no significant differences observed in female age, body mass BMI, AFC, basal E_2_ and FSH levels, or total doses of HMG and HCG, infertile year and gravidity (all *P* > 0.05, SMD< 0.1; **Table 1**).

**Table 1:** Characteristics of IVF/ICSI cycle population.

Variables	Control(n=160)	Survivor (n=32)	SMD	Control(n=160) IPTW	Survivor (n=32) IPTW	SMD IPTW	β IPTW	CI_95 IPTW	*P* IPTW
Age (y)	32.78 ± 0.35	36.06 ± 0.90	0.68	34.00 ± 0.57	34.10 ± 1.02	<0.1	0.01	[-0.107, 0.117]	0.93
BMI (kg/m2)	22.19 ± 0.17	22.43 ± 0.50	0.10	22.07 ± 0.20	22.15 ± 0.61	<0.1	0.02	[-0.226, 0.255]	0.90
FSH (mIU/ml)	5.90 ± 0.16	5.55 ± 0.38	0.17	5.66 ± 0.22	5.72 ± 0.29	<0.1	0.02	[-0.228, 0.267]	0.88
E2 (pg/ml)	42.39 ± 2.66	36.78 ± 2.62	0.22	39.28 ± 2.70	40.34 ± 4.55	<0.1	0.00	[-0.017, 0.021]	0.84
AFC (n)	11.34 ± 0.48	6.06 ± 1.18	0.83	9.51 ± 0.79	8.85 ± 2.12	<0.1	-0.01	[-0.114, 0.086]	0.78
HMG (IU)	1907.81 ± 50.86	1746.09 ± 116.96	0.25	1866.12 ± 99.63	1859.26 ± 134.41	<0.1	0.00	[-0.001, 0.001]	0.97
HCG (IU)	1939.38 ± 143.49	2031.25 ± 289.20	0.05	1889.96 ± 169.34	1911.86 ± 408.95	<0.1	0.00	[0.000, 0.000]	0.96
Infertile Year (y)	4.18 ± 0.22	3.48 ± 0.47	0.26	4.00 ± 0.26	3.97 ± 0.78	<0.1	0.00	[-0.231, 0.223]	0.97
Gravidity (n)	0.96 ± 0.10	1.38 ± 0.26	0.30	1.14 ± 0.19	1.20 ± 0.24	<0.1	0.04	[-0.354, 0.425]	0.86
Male factor (%)	73 (45.6%)	11 (34.4%)	0.11	42.1% ± 5.41	43.6% ± 13.81	<0.1	0.06	[-1.130, 1.253]	0.92
Tubal factor (%)	37 (23.1%)	9 (28.1%)	0.05	26.6% ± 5.99	23.6% ± 11.23	<0.1	-0.16	[-1.533, 1.209]	0.82
Uterine factor (%)	20 (12.5%)	3 (9.4%)	0.03	8.8% ± 2.05	9.2% ± 5.87	<0.1	0.05	[-1.427, 1.529]	0.94
PCOS (%)	18 (11.2%)	5 (15.6%)	0.04	11.1% ± 3.54	11.6% ± 6.85	<0.1	0.06	[-1.440, 1.545]	0.94
Other factors (%)	12 (7.5%)	4 (12.5%)	0.05	11.5% ± 4.16	12.0% ± 9.24	<0.1	0.06	[-1.852, 1.957]	0.96

Values are mean ± SEM. There were no statistically significant differences in any of the parameters between the survivor and control groups.

The distribution of cancer types in the survivor group is presented in **Table 2**, with reproductive system malignancies being the most prevalent. The other group included osteosarcoma, lung cancer, liver cancer, gallbladder cancer, and lymphoma. Ovarian stimulation protocols used in the two groups are detailed in **Supplementary Table 1**. The ovarian stimulation protocols were comparable between the cancer survivor group and the control group, with no significant differences observed.

**Table 2:** Type of malignancy in women undergoing IVF.

Type of cancer	No. of patients (n)	Chemotherapy (n)	Radiotherapy (n)
Gestational trophoblastic	11	11	0
Breast cancer	6	5	3
Other	6	6	1
Nasopharyngeal	5	3	5
Brain	2	2	1
Thyroid	1	0	1
Cervical	1	1	0

### IVF outcomes

IVF outcomes were summarized in **Table 3**. Compared with controls, women with a history of chemotherapy or radiotherapy had a significantly lower number of MII oocytes (β=-3.06 95%CI [-5.568, -0.555], *P* = 0.02), and a reduced high-quality embryo rate (β=-0.25 95%CI [-0.375, -0.128], *P* < 0.001). No significant differences were observed between the two groups in the number of retrieved oocytes, oocytes retrieval rate, fertilization rate, number of embryos, or number of blastocysts (all *P* > 0.05).

**Table 3:** IVF outcomes in survivor and control group.

Variables	Control (n=160)	Survivor (n=32)	β	95%CI	P	Adj β	Adj 95%CI	Adj P
Number of punctured follicles (n)	11.66 ± 0.79	8.91 ± 1.32	-2.74	[-5.769, 0.283]	0.08	-2.71	[-5.406, 0.013]	0.06
Rate of oocytes retrieved (%)	0.72 ± 0.02	0.78 ± 0.05	0.07	[-0.046, 0.177]	0.25	0.07	[-0.010, 0.145]	0.09
No. Of MII oocytes (n)	10.30 ± 0.72	7.22 ± 1.15	-3.09	[-5.764, -0.411]	0.02	-3.06	[-5.568, -0.555]	0.02*
Fertilization rate (%)	0.83 ± 0.02	0.73 ± 0.06	-0.10	[-0.221, 0.023]	0.11	-0.10	[-0.206, 0.008]	0.07
No. Of embryos (n)	7.56 ± 0.55	6.01 ± 0.88	-1.54	[-3.588, 0.505]	0.14	-1.52	[-3.300, 0.255]	0.09
High quality embryos (%)	5.34 ± 0.36	3.91 ± 0.80	-1.42	[-3.147, 0.303]	0.11	-1.41	[-2.760, -0.064]	0.04*
Rate of high-quality embryos (%)	0.80 ± 0.02	0.55 ± 0.07	-0.25	[-0.402, -0.100]	0.00	-0.25	[-0.375, -0.128]	<0.01*
No. Of blastocyst (n)	0.59 ± 0.10	0.64 ± 0.19	0.05	[-0.368, 0.469]	0.81	0.05	[-0.322, 0.426]	0.78

Values are mean ± SEM.*P<0.05.

Subsequently, we analyzed the association between cancer type and IVF outcomes and found that cancer type was not significantly associated with any IVF outcome (**Supplementary Table 2**).

### Factors associated with high-quality embryo rate

Subsequently, we performed a multivariate regression analysis on factors potentially influencing the high-quality embryo rate. We found that high-quality embryo rate was found to be associated with cancer treatment modality. And a multivariate analysis was further performed in the survivor group. No significant associations were identified between the high-quality embryo rate and cancer treatment, treatment-to-ART interval, BMI, FSH, age, or E_2_. (**Supplementary Table 3**). To assess potential multicollinearity among the variables included in the multivariable regression model, variance inflation factor (VIF) analysis was performed. The VIF values for all included predictors were low, ranging from 1.12 to 2.28 (treatment: 1.63, interval: 1.70, age: 1.28, BMI: 1.12, FSH: 1.14, and E2: 2.28), indicating no evidence of significant multicollinearity among the covariates.

To explore whether ART outcomes are related to the interval after chemotherapy or radiotherapy. We further evaluated the relationship between IVF outcomes and the interval between cancer treatment and ART initiation. No significant associations were observed between interval duration and any IVF outcome (**Supplementary Table 4**).

### Pregnancy outcomes after FET

Among patients who proceeded to FET, baseline characteristics were comparable between survivors and controls (**Table 4**). After adjustment for age, BMI, gravidity, endometrial thickness, ovarian stimulation protocol, and cancer type, no significant differences were found in IVF outcomes between the survivor and control groups. Consistently, no significant differences in CPR or CLBR were observed among two groups (CPR: HR = 1.18 95%CI [0.59,1.38] *P* = 0.62; CLBR: HR = 0.96 95%CI [0.43,2.16] *P* = 0.91) (**Supplementary Figure 1**). We analyzed the implantation rate (OR = 1.32, 95%CI [0.51-3.45], P = 0.56) and biochemical pregnancy rate (OR = 1.51, 95%CI[0.41-5.65], P = 0.53), and found no significant differences between the two groups. The spontaneous abortion rate was 6.25% (2/32) in Survivor and 4.23% (11/260) in Control, with no significant difference between groups (Fisher’s exact test, OR = 1.51, 95% CI 0.32–7.08, *P* = 0.65).

**Table 4:** Characteristics of FET population.

Variables	Control(n=260)	Survivor (n=32)	SMD	Control(n=260) IPTW	Survivor (n=32) IPTW	SMD IPTW	β	95% CI IPTW	*P* IPTW
Age (y)	33.10 ± 0.24	35.94 ± 0.99	0.58	34.40 ± 0.61	34.55 ± 1.25	<0.1	0.01	[-0.100, 0.111]	0.92
BMI (kg/m2)	22.13 ± 0.14	22.34 ± 0.43	0.09	22.28 ± 0.16	22.30 ± 0.42	<0.1	0.01	[-0.187, 0.199]	0.95
Infertile Year (y)	4.13 ± 0.16	2.48 ± 0.40	0.67	3.71 ± 0.18	3.65 ± 0.67	<0.1	0.01	[-0.214, 0.198]	0.94
Pregnant Time (n)	0.86 ± 0.07	1.64 ± 0.28	0.55	1.38 ± 0.21	1.33 ± 0.34	<0.1	0.02	[-0.371, 0.327]	0.90
FSH (mIU/ml)	4.98 ± 0.15	5.92 ± 0.80	0.25	5.33 ± 0.24	5.42 ± 0.49	<0.1	0.01	[-0.148, 0.175]	0.87
E2 (pg/ml)	46.62 ± 3.17	39.23 ± 7.78	0.15	47.61 ± 4.19	49.26 ± 14.07	<0.1	0.00	[-0.009, 0.010]	0.91
Endometrial Thickness (mm)	10.35 ± 0.15	10.40 ± 0.40	0.02	10.62 ± 0.19	10.62 ± 0.64	<0.1	0.00	[-0.210, 0.211]	0.99
Number of embryos transferred (n)	1.85 ± 0.02	1.70 ± 0.08	0.16	1.81 ± 0.04	1.80 ± 0.08	<0.1	0.07	[-1.199, 1.052]	0.90
Number of high-quality embryos transferred (n)	0.01 ± 0.01	0.09 ± 0.07	0.08	0.11 ± 0.10	0.11 ± 0.11	<0.1	0.00	[-0.289, 0.290]	0.99
Hormone replacement therapy (%)	131 (50.4%)	13 (39.4%)	0.11	43.9% ± 4.21	44.0% ± 11.40	<0.1	0.00	[-0.967, 0.975]	0.99
Mild stimulation (%)	77 (29.6%)	13 (39.4%)	0.10	30.7% ± 4.07	29.1% ± 10.44	<0.1	0.08	[-1.142, 0.988]	0.89
Natural cycle (%)	52 (20.0%)	7 (21.2%)	0.01	25.3% ± 5.17	26.9% ± 10.36	<0.1	0.08	[-1.090, 1.248]	0.89

mean ± SEM.

## Discussion

Advances in early cancer detection and more effective therapies have markedly improved survival among women of reproductive age, making fertility and post-treatment reproductive outcomes increasingly important. For many young survivors, the ability to conceive is a key component of quality of life. Assisted reproductive technology, particularly IVF-ET, therefore plays a crucial role in helping cancer survivors achieve pregnancy, highlighting the need to better define ART outcomes in this population.

Our study provides evidence that previous chemotherapy and/or radiotherapy exposure may be associated with specific aspects of ART outcomes beyond the reduction in ovarian reserve. Despite comparable ovarian reservation between cancer survivors and controls, survivors exhibited fewer MII oocytes and a lower high-quality embryo rate, suggesting that the impact of anticancer therapy may extend beyond quantitative follicle depletion and potentially influence oocyte developmental competence. However, the clinical implications of these findings require careful interpretation, as the ability to achieve pregnancy ultimately depends on multiple factors, including embryo competence and endometrial receptivity.

However, previous studies have reported no significant differences in oocyte yield or fertilization rates between women with and without a history of cancer. Das et al. conducted a study involving 41 women with cancer and 48 control women, showing that cancer diagnosis itself did not adversely affect ovarian response among women undergoing fertility preservation before gonadotoxic treatment (4). However, this study excluded patients who had already received chemotherapy or radio therapy. Another large-scale study including 587 women with a history of cancer and 34,203 women without such a history found no significant differences between the two groups in the number of oocytes retrieved (aRR: 0.95 (0.89–1.02)) or in the proportion of autologous oocytes fertilized (aRR: 0.99 (0.95–1.03)) between two groups (14). However, these studies did not specifically distinguish patients who had undergone chemotherapy or radiotherapy, nor did they control for baseline ovarian reserve. As a result, the potential impact of anticancer treatments on oocyte quality may have been underestimated.

Luke’s study included 53,426 women; 441 women were diagnosed with cancer within 5 years prior to ART start found that among women using only autologous oocytes, women with cancer overall were significantly less likely to become pregnant and to have a live birth (15). However, none of these studies specifically compared patients who had undergone chemotherapy or radiotherapy as a distinct group. Dillon’s prospective cohort study included 84 female survivors with chemotherapy and 98 similar-aged controls confirmed that survivors have diminished AFC (16). Barton’s research (11) demonstrated that patients who had received chemotherapy or radiotherapy had significantly fewer oocytes retrieved. However, their study did not provide a detailed analysis of blastocyst number, or proportion of high-quality embryos and also did not match the AFC number between two groups.

In our research we matched the two groups of people with AFC and we observed a significantly lower MII oocyte number and in the survivor group, while the blastocyst number showed no significant difference compared to the control group. This indicates that chemotherapy or radiotherapy may associated with lower oocyte maturation independently of ovarian reserve, as comparable AFC values were observed between groups.

Although chemotherapy and radiotherapy have been suggested to negatively affect oocyte developmental competence through mitochondrial dysfunction, endoplasmic reticulum (ER) stress (17, 18), oxidative damage, and DNA injury (19, 20), the long-term impact of cancer treatment on embryo quality in patients undergoing assisted reproductive technology remains unclear. Previous studies have reported impaired embryo developmental potential after gonadotoxic treatments (10, 21). However, our multivariable analysis demonstrated that cancer treatment type was not associated with high-quality embryo rate after adjustment for age, BMI, FSH, E2, and treatment interval. The potential effects of chemotherapy or radiotherapy on embryo developmental competence may be modulated by individual susceptibility. Besides, the relatively limited sample size of our cohort may have reduced the statistical power to detect modest effects of cancer treatment on embryo quality. Therefore, cancer treatment history alone may not fully predict embryo quality among patients undergoing fertility treatment. Further large-scale studies incorporating detailed treatment information and molecular biomarkers of oocyte quality are warranted to better elucidate the reproductive consequences of cancer therapy.

This study found no differences in IVF/ICSI outcomes between different interval year. Studies have shown that ovarian function can recover as the interval since treatment increases, but it declines with increasing age (22, 23). FERNáNDEZ’s research found that for individuals under 35 years old, the number of oocytes does not decrease with increasing time since the end of treatment. However, for those over 35, the average number of oocytes collected decreases as the interval between the end of cancer treatment and the first oocyte retrieval increases (24). This can explain the results of our study analyzing different interval times. However, this lack of significant association should not be interpreted as definitive evidence that the time interval has no impact on reproductive outcomes. Due to the limited sample size, the analysis may have lacked statistical power to detect subtle, non-linear, or treatment-specific associations. Therefore, our conclusions are limited to stating that no significant correlation was identified within the analyzed cohort, and further studies with larger populations are warranted.

Achieving clinical pregnancy also heavily depends on implantation success (25). In this study, although cancer survivors exhibited a lower number of mature oocytes and a reduced proportion of high-quality embryos during ovarian stimulation, no significant differences were observed in CPR or CLBR following FET cycles compared with controls. Similar to our study, Elkin’s research found that there were no differences in terms of pregnancy (55.7%vs.54.7%), implantation (39.8%vs.38.2%), miscarriage (29.5%vs.26.9%), or delivery rates (39.3%vs.39.9%) between the unexposed group and the patient, respectively (26). These findings suggest that the potential adverse effects of anticancer treatment on ART outcomes may primarily occur during the ovarian stimulation and embryo generation stages. Once viable embryos are successfully obtained and selected for transfer, their implantation potential and subsequent pregnancy development may not be substantially impaired. The success of FET cycles largely depends on embryo developmental competence and endometrial receptivity. Consistent with our findings, previous studies have suggested that exposure to chemotherapy or radiotherapy may not substantially compromise uterine receptivity-related processes, including early blastocyst attachment and trophoblast invasion. (27, 28). While encouraging, this result should not be construed as definitive confirmation of preserved endometrial receptivity. Given the relatively small number of FET cycles and the resulting wide confidence intervals may reflect limited statistical precision.

In comparison to prior related studies, our study exhibits several strengths. First, we excluded individuals with ovarian surgeries from both groups, so we minimize potential confounding effects of ovarian dysfunction resulting from mechanical ovarian injury on the outcomes. Second, we performed IPTW, which allowed us to investigate whether ART outcomes in patients who underwent chemotherapy or radiotherapy are still affected when their ovarian reserve is comparable to that of non-cancer patients. And we also performed a multivariate analysis to explore the potential factors contributing to the decreased rate of high-quality embryos in survivor group. Finally, we analyzed whether there exist differences in IVF/ICSI and FET outcomes between long time interval and short time interval group, providing insights that may inform future prospective research design and assist in patient fertility counseling.

However, this study also has several limitations. First, due to the retrospective design, selection bias and residual confounding cannot be completely excluded despite adjustment for important clinical variables and the use of IPTW analysis and the relatively small number of cancer survivors may have limited statistical power to detect modest differences in clinical outcomes such as pregnancy and live birth rates. Second, the survivor group included patients with various cancer types and anticancer treatment regimens, which may differ in their gonadotoxic potential. However, the relatively small sample size prevented further stratified analyses according to specific cancer types, chemotherapy agents, or radiotherapy characteristics. Third, although we adjusted for major ovarian reserve-related factors and other important clinical characteristics, some potential confounders, including lifestyle factors (such as smoking, alcohol consumption, and diet), socioeconomic status, family genetic background, and detailed treatment intensity, were not available in our retrospective database and may have influenced ART outcomes. Finally, as this was a single-center retrospective cohort study, the generalizability of our findings requires further validation in larger multicenter prospective studies.

## Conclusion

In conclusion, this study demonstrated that women with a history of chemotherapy or radiotherapy and non-cancer women have comparable ovarian function outcomes exhibiting reduced oocyte maturity, and lower high-quality embryo rate in IVF/ICSI cycle. There was no difference in FET outcomes between survivor and control group and no statistically significant associations between interval and FET clinical outcomes were observed in the present cohort.

## Data sharing statement

The raw clinical data generated and analyzed during this study are not publicly available due to privacy and ethical restrictions. Researchers with a legitimate scientific interest may contact the corresponding author to request data upon reasonable request.

## Data Availability

The raw clinical data generated and analyzed during this study are not publicly available due to privacy and ethical restrictions. Researchers with a legitimate scientific interest may contact the corresponding author to request data upon reasonable request. Requests to access the datasets should be directed to Zhouxq1999@163.com.
