# Supplementary material for: IVF outcomes in cancer survivors: lower ovarian response and early embryo development but comparable success in frozen embryo transfer cycles — a retrospective cohort study
Source: Front Endocrinol (Lausanne). 2026 Aug 26;17:1898170. doi: 10.3389/fendo.2026.1898170 (PMC13553496; doi:10.3389/fendo.2026.1898170)
Supplement: Supplementary file 1 [file SupplementaryFile1.pdf]

Sup Table 1 Ovulation-inducing Treatments In Two Groups

Ovulation-inducing Treatments	Survivor (n=32)	Control (n=160)	*P* Value
Progestin-primed Ovarian Stimulation	18	93	0.85
GnRH-ant	5	29	0.81
Mild stimulation	6	20	0.38
Long Protocol	1	6	1.00
Short Protocol	1	1	0.32
Late-start Protocol	0	4	1.00
Natural Cycle	1	1	0.32
Ultra-long protocol	0	6	1.00

Note: * = P＜0.05.

Sup Table 2 Association between cancer type and IVF outcomes

Description	F_Statistic	*P*	Adj_R2
Number of punctured follicles (n)	1.497	0.2381	0.255
Oocytes retrieved (n)	1.173	0.3656	0.223
Rate of oocytes retrieved (%)	0.515	0.7888	-0.208
No. Of MII oocytes (n)	2.21	0.0928	0.214
Fertilization rate (%)	0.749	0.6187	-0.144
No. Of embryos (n)	1.389	0.275	0.25
High quality embryos (n)	1.74	0.1721	0.244
Rate of high-quality embryos (%)	0.294	0.9313	-0.112
No. Of blastocyst (n)	0.336	0.9082	-0.251

Note: F statistics (representing the overall significance of the model), P values, and adjusted R^2 values (indicating the proportion of variance explained by the model) are presented.

Sup Table 3 Multivariable analysis about high-quality embryo rate

Variable	β	95% CI	P value
Radiotherapy	0.014	-0.585 - 0.613	0.96
Chemotherapy	0.198	-0.191 - 0.587	0.30
Interval	-0.001	-0.002 - 0.002	0.94
BMI	0.002	-0.061 - 0.041	0.68
FSH	-0.060	-0.122 -0.003	0.06
Age	0.012	-0.025 - 0.031	0.85
E2	-0.002	-0.015 - 0.011	0.68

Note: * = P＜0.05

Sup Table 4 Association between the interval from cancer treatment to IVF and IVF outcomes

Outcome	Beta	Standard Error	95%CI Lower	95%CI Upper	*P*
Number of punctured follicles (n)	-0.0091	0.0213	-0.0612	0.0430	0.68
Rate of oocytes retrieved (%)	0.0000	0.0020	-0.0048	0.0049	0.98
No. Of MII oocytes (n)	0.0067	0.0277	-0.0612	0.0745	0.82
Fertilization rate (%)	-0.0001	0.0012	-0.0031	0.0029	0.95
No. Of embryos (n)	0.0107	0.0251	-0.0507	0.0721	0.68
High-quality embryos (%)	0.0105	0.0207	-0.0401	0.0612	0.63
Rate of high-quality embryos (%)	0.0024	0.0029	-0.0048	0.0095	0.44
No. Of blastocyst (n)	-0.0001	0.0041	-0.0101	0.0099	0.98

Note: Multivariable linear regression models were performed to evaluate the association between cancer type and IVF outcomes. Beta coefficients (β) represent the estimated change in each IVF outcome associated with cancer type. Standard errors (SE) indicate the variability of the estimated β coefficients. The 95% confidence interval (CI) represents the range of values within which the true effect estimate is expected to lie with 95% confidence.

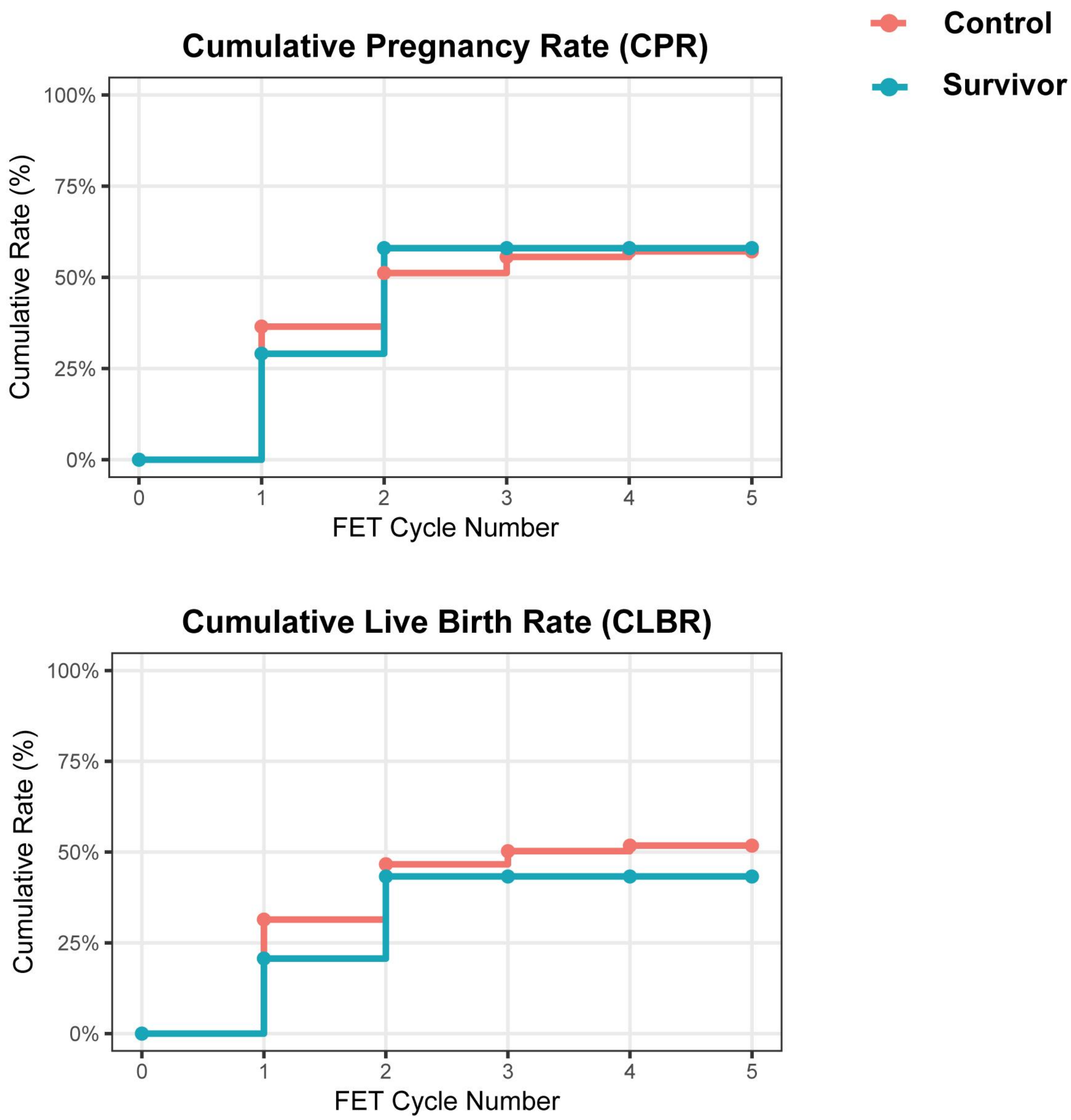

Sup Fig 1 Cumulative Pregnancy Rate and Cumulative Live Birth Rate by FET Cycle
